# Cell-Based Therapies: Ferromagnetic Versus Superparamagnetic Cell Targeting

**DOI:** 10.3390/bioengineering12060657

**Published:** 2025-06-16

**Authors:** Tasneem Halhouli, Lisa Münchhalfen, Sarkawt Hamad, Larissa Schmitz-Ullrich, Frank Nitsche, Felix Gaedke, Astrid Schauss, Linlin Zhang, Quoc-Khanh Pham, Gang Bao, Kurt Paul Pfannkuche

**Affiliations:** 1 Center for Physiology and Pathophysiology, Institute for Neurophysiology, University of Cologne, Medical Faculty and University Hospital of Cologne, 50931 Cologne, Germany; 2 Marga-and-Walter-Boll Laboratory for Cardiac Tissue Engineering, University of Cologne, 50931 Cologne, Germany; 3 Biology Department, Faculty of Science, Soran University, Soran 44008, Kurdistan Region, Iraq; 4 Institute for Zoology, General Ecology, University of Cologne, 50674 Cologne, Germany; 5 Excellence Cluster on Cellular Stress Responses in Aging-Associated Diseases (CECAD), Imaging Facility, 50931 Cologne, Germany; 6 Department of Bioengineering, Rice University, Houston, TX 77030, USA; 7 Department of Pediatric Cardiology, University Hospital of Cologne, 50937 Cologne, Germany; 8 Center for Molecular Medicine Cologne (CMMC), University of Cologne, 50931 Cologne, Germany

**Keywords:** superparamagnetic iron oxide nanoparticles (SPIONs), ferromagnetic particles, murine MSCs

## Abstract

Stem-cell-based therapies rely on the transplantation of stem cells or stem-cell-derived organotypic cells into injured tissues in order to improve or restore tissue function that has been impaired by various diseases. The potential of induced pluripotent stem cells has created many applications in the field of cell therapy, for example. Some applications, for example, those in cardiac cell therapy, suffer from low or very low efficiencies of cell engraftment. Therefore, magnetic cell targeting can be discussed as a method for capturing superparamagnetic nanoparticle-labelled cells in the tissue. Here, we employ superparamagnetic iron oxide nanoparticles (SPIONs) for the intracellular magnetic loading of mesenchymal stem cells (MSCs). In addition, we test a novel strategy of labelling MSCs with ferromagnetic particles. The adhesion assays demonstrate a faster adhesion kinetic of SPIONs-loaded MSC spheroids when a magnetic field was applied, resulting in >50% spheroid adhesion after 30 min. Clustering of cells inside the magnetic field is a second potential mechanism of magnetic cell retention and >80% of cells were found to be aggregated in clusters when placed in a magnetic field for 10 min. SPIONs-loaded and ferromagnetic-particle-loaded cells performed equally in the cell clustering assay. In conclusion, the clustering of SPION-labelled cells explains the observation that magnetic targeting reaches maximal efficiency in vivo after only 10 min of magnetic field application. This has significant implications for magnetic-targeting-assisted stem cell and cell replacement therapies.

## 1. Introduction

Cell replacement therapies often face the issue of low cellular retention. Even though in vivo studies confirm that cell transplantation can replace and improve the function of damaged tissue, rapid diffusion and limited survival rates after cell injection are the reason for low engraftment rates [1,2,3,4].

To overcome low cellular retention, mainly in the field of cardiac cell therapy, reports of magnetic targeting and application of an external magnetic field show promising results; however, the overall efficiency remains often low, and the underlying mechanism of enhanced cell engraftment remains unclear [1,5,6,7,8,9]. The efficiency of cell engraftment depends on the type of cell included in a study; while intramyocardial transplantation of cardiomyocytes usually results in poor cell engraftment rates, magnetically assisted transplantation of cardiac myofibroblasts engineered to express connexin 43 resulted in very high (30% of injected cells) engraftment rates [10]. Other studies indicate that cell therapies with microtissues (i.e., assemblies of organotypic cells to a functional tissue in mm scale) and/or 3D spheroids (i.e., microtissues characterized by a regular spherical shape) can enhance engraftments. But these approaches still struggle with the same problem of low retention, even when adding fast-adhesive cells, e.g., mesenchymal stem/stromal cells (MSCs) [2,3,6,11,12].

Magnetically labelling cells with superparamagnetic iron oxide nanoparticles (SPIONs) or magnetic iron oxide nanoparticles (MIONs) through magnetic-force-mediated endocytosis is described well in the work of Zhang [13]. Iron oxide nanoparticles with diameters below 20 nm hold superparamagnetic properties and are highly biocompatible [5,13]. Therefore, SPIONs made from iron oxide are suitable candidates for exerting magnetic forces on cells. Potential applications that have been tested in vivo include the targeting of endothelial cells to the site of large artery injury [14], the targeting of mesenchymal stem cells to improve sphincter structure in a model of urinary incontinence [15], the targeting of endodermal progenitors to the liver [16], and the targeting of cells to the brain after experimental disruption of the blood–brain barrier [17]. Targeting of embryonic and pluripotent stem-cell-derived cardiomyocytes to the heart showed a 7-fold increase in cell retention 2 weeks after transplantation [18]. Taken together, these and other studies suggest an increased engraftment of magnetically loaded cells when an external magnet is applied.

In this study, we show the feasibility of magnetically targeting murine mesenchymal stem cells through SPIONs loading and a second method of ferromagnetic particle labelling. We propose two potential mechanisms of action by which magnetic targeting could enhance cell retention. Firstly, it is postulated that the attachment of magnetically labelled cells to an extracellular matrix can be improved by applying magnetic force. A faster and/or closer contact of the cells to the matrix and an increased dwell time will contribute to this effect. Secondly, magnetic forces could result in a forced aggregation of cells, thereby reducing wash out or cell death mediated by lack of cell–cell interactions. Based on the assumption that forced aggregation could improve persistence of cells, we hypothesize that ferromagnetic labelling of cells could outcompete SPIONs, because ferromagnetic particles become permanently magnetised during exposure to a strong magnetic field. This could result in a fast and stable aggregation of cells even when the external field is removed. This hypothesis is based on the fact that ferromagnetic particles, unlike superparamagnetic particles, generate magnetic forces that stabilize the cluster in the absence of an external magnetic field.

## 2. Methods

### 2.1. Mesenchymal Stem Cell Culture

Murine mesenchymal stem cells (mMSCs) were isolated from the bone marrow of 6–8-week-old male mice (129S2), as described previously [11]. In short, the mouse was sacrificed by cervical dislocation, the femur bones were isolated, and the bone marrow was flushed with DMEM using a syringe with an injection cannula. The flushed cell suspension was filtered and plated on 10 cm culture dishes inside DMEM high glucose (4.5 g/L) (Gibco by Thermo Fisher, Karlsruhe, Germany #21885108), supplemented with 15% (*v*/*v*) foetal bovine serum (FBS, Sigma-Aldrich, Darmstadt, Germany #F9665) and Primocin antimicrobial agent for primary cell culture (InvivoGen, Tolouse, France, #ant-pm-05), and incubated at 37 °C with 5% CO_2_ in a humidified incubator. Non-adherent cells were removed after 12 h culturing. When the cell culture reached 80–90% confluence of attached cells, the cells were sub-cultured using 0.05% trypsin–EDTA solution (Gibco, #25300054). The mMSC culture medium was DMEM low glucose (1 g/L) (Gibco, #11885084) supplemented with 15% (*v/v*) FBS, 1x non-essential amino acids (Gibco, #11140050), 100 µM 2-mercaptoethanol (Gibco, #31350010), and 6 ng/mL fibroblast growth factor 2 (Peprotech by Thermo Fisher, Karlsruhe, Germany # 100-18B); mMSCs were incubated in a humidified incubator at 37 °C, with 5% CO_2_.

### 2.2. Synthesis of Magnetic Nanoparticles

Phospholipid–PEG-coated magnetic iron oxide nanoparticles were synthesized in two steps. First, magnetite nanocrystals were synthesized by the thermal decomposition of Tris(acetonylacetonato)-iron(III) (Fe(acac)3) according to previous publications [13,19]. The nanocrystals were dispersed in either toluene (10 and 15 nm) or chloroform (19 and 25 nm). Next, water-dispersible magnetic nanoparticles were obtained by coating these hydrophobic nanocrystals with amphiphilic DSPE-mPEG copolymers using a dual-solvent exchange method. For example, to coat 15 nm nanocrystals, 5 mg of nanocrystals, 14 mg of DSPE-mPEG, and 1.12 mg of LysoPC were mixed in 2 mL of chloroform, and 20 mL of DMSO was added dropwise to the mixture with gentle shaking. After toluene and chloroform were removed by evaporation under a vacuum, 40 mL of distilled water was slowly added to the mixture and DMSO was removed by solvent exchange with Vivaspin centrifugal filter tubes (MW = 100 kDa). To remove empty micelles formed by DSPE-mPEG and LysoPC, the solution was centrifuged twice (80,000× *g*, 4 °C and 1 h) and the supernatant was discarded. After centrifugation, coated nanoparticles were dispersed in distilled water. For 10, 19, and 25 nm nanocrystals, the weights of nanocrystals were changed to 3.7, 6.3, and 8.3 mg, respectively, to keep the total surface area of the nanocrystals constant during coating. The coating procedure and characterization has been described in detail before [20].

To characterize the magnetic properties of the nanocrystals, the nanocrystals dispersed in toluene were precipitated with ethanol and the pellets were dried by an argon beam. Magnetisation of the collected powder was measured at room temperature with a superconducting quantum interference device (SQUID). The hydrodynamic diameter of the magnetic nanoparticles was measured by dynamic light scattering (DynaPro Nanostar, Wyatt Technology, Santa Barbara, CA, USA). The mass-weighted diameter and polydispersity index were reported.

### 2.3. Intracellular Loading of Superparamagnetic Iron Oxide Nanoparticles (SPIONs)

SPIONs with a particle size of 15 nm were made using Fe_3_O_4_ nanoparticles with a PEG coating. Separate batches of SPIONs with PEG and 1,1′-Dioctadecyl-3,3,3′,3′-Tetramethylindocarbocyaninperchlorat (Dil) coating, which can be detected at a wavelength of 546 nm, were produced in the laboratory of Gang Bao (Department of Bioengineering, Rice University, Houston, TX 77030, USA), as described in [13].

For loading MSCs with SPIONs, cells were co-incubated overnight with 60 µg/mL SPIONs-supplemented medium. The incubator was equipped with a plate magnet under the culture dish for magnetic-force-mediated endocytosis (+MF) or without placing a magnet under the culture dish (−MF). The cultures were maintained overnight inside a humidified incubator at 37 °C and 5% CO_2_. Intracellular loading success was confirmed by flow cytometry, fluorescence microscopy, and transmission electron microscopy (TEM).

### 2.4. Quantification of Iron Content of Cells Through a Ferrozine-Based Assay

Quantification of intracellular iron oxide was measured by a ferrozine-based assay following Zhang (2023) [13]. Briefly, SPION-loaded cells were detached from the culture plate, evacuated overnight, and iron was released from the cells by adding 12 M hydrochloric acid (Sigma-Aldrich, Taufkirchen, Germany, 339253-100ML), 8 M sodium hydroxide solution (Sigma-Aldrich, 72068-100ML), 4 M ammonium acetate (Sigma-Aldrich, #A2706-100ML), 5% (*w*/*w*) hydroxylamine hydrochloride (Sigma-Aldrich, #431362-50G), and H_2_O. Ferrozine of 0.1% (*w*/*w*) concentration (Sigma-Aldrich, #82950-1G) was added to form a ferrous–ferrozine complex, and the formation of the complex was determined at 562 nm absorbance. The unknown sample iron concentration was calculated through the slope of an iron standard curve. The iron standard (Sigma-Aldrich, #43149-100ML-F) was prepared in a dilution series from 0 to 500 µg/mL in water and treated similarly with ammonium acetate, hydroxylamine hydrochloride, and H_2_O, and each standard concentration was determined at 562 nm absorbance after adding 0.1% (*w*/*w*) ferrozine.

### 2.5. SPIONS Cytotoxicity Assay

Following ISO-Norm 10993-5, the cytotoxicity of SPIONs in vitro was evaluated by observing morphology, proliferation, and viability for three days. mMSCs were seeded and loaded with PEG-coated SPIONs and stained at three different time points (24, 48, and 72 h) with Hoechst33342 (Thermo Fisher, #62249), propidium iodide (Invitrogen by Thermo Fisher, #P3566), and fluorescein diacetate (Invitrogen, #F1303) to determine the alive–dead ratio and morphology of the mMSCs.

### 2.6. Cell Surface Magnetic Targeting Using Anti-CD29

Ferromagnetic particles with diameters of 2 µm were bio-linked to MSCs through the surface receptor Integrin beta-1. The expression of CD29 on the surface of the mMSCs was determined with flow cytometry using anti-CD29–Biotin (Miltenyi Biotec, Bergisch Gladbach, Germany #130-101-943) and a secondary Streptavidin–Alexa Flour 488 (BioLegend, San Diego, CA, USA #405235) antibody.

For magnetically targeting the mMSC cell surface, 0.25 × 10^6^ mMSCs were firstly incubated 1:50 with anti-CD29 antibody conjugated with Biotin (Miltenyi, #130-101-943) for 30 min at 4 °C, followed by incubation with 1:10 SPHERO Streptavidin Ferromagnetic Particles (Spherotech, Lake Forest, IL, USA #SPH-SVM-80-5) and 1:1000 Hoechst 33342 (Thermo Fischer, #H3570) for 30 min at 4 °C. With a Streptavidin–Alexa Flour 488 stain (BioLegend, #405235), the CD29–Biotin-conjugated antibodies were visualized for fluorescence microscopy.

### 2.7. Generation of 3D Cellular Spheroids by Hanging Drop Technique

Hanging drop mMSC spheroids were formed with a density of 800 cells per 20 µL drop in mMSC culture medium. Cell suspension drops were placed on the inner surface of a culture dish lid and incubated for two days. For SPION-loaded mMSC spheroids, cells were loaded with SPIONs using magnetic-force-mediated endocytosis prior to spheroid formation.

Ferromagnetic-labelled spheroids were formed by co-incubating 60 µg/mL ferromagnetic particles with mMSCs on a shaker incubator for 5 min before drop formation.

### 2.8. Adhesion Test of Magnetically Labelled Spheroids on Collagen

Cell culture plates were coated with 50 µg/mL collagen type I rat tail (Sigma-Aldrich, #08-115) and SPIONs and ferromagnetic particle mMSC spheroids were tested for their adhesion time for a period of 2 h against an unlabelled control group. Coated plates were placed on top of a magnet. The adhesion of spheroids was checked at the following time points: 0, 15, 20, 30, 35, 45, 60, 75, 90, 105, and 120 min. At each time point, one plate was transferred from the incubator to the microscope and the number of spheroids was counted before and after removing the medium from the plate. To count the attached spheroids, 1 mL medium was added to the plate slowly and under the microscope and only the non-moving spheroids were counted as attached spheroids.

### 2.9. Magnetically Labelled Single Cells Aggregation and Clumping

For the aggregation of magnetically targeted single cells, a Nd permanent magnet with a conic iron tip (the magnetic field close to the conic tip is measured with a Gauss meter determined as 150 mT) was applied in the proximity of a single cell suspension. This work was carried out in an Eppendorf tube. The tip magnet was applied for 1, 5, or 15 min at room temperature. The formed clusters were removed gently with a micropipette and placed onto a glass slide for fluorescence microscopy or in a microtube for DNA extraction.

### 2.10. Quantification of Magnetically Induced Cell Clumps

After the aggregation of single cells with a tip magnet, cell clumps were quantified through nucleic acid concentration. For DNA extraction, the DNEasy DNA extraction kit (Qiagen, Hilden, Germany #69506) was used, following the manufacturer’s protocol. DNA concentration was measured with absorbance at 260 nm with a nanodrop spectrophotometer (peqLab, Erlangen, Germany #ND1000). A standard curve of nucleic acid concentration of fixed cell numbers was performed before, measuring the DNA concentration of formed cell clumps.

### 2.11. Scanning Electron Microscopy (SEM)

Cell cultures were fixed with cacodylate-buffered glutaraldehyde (3%) at 4 °C for 120 min followed by post-fixation with 1% osmium tetroxide for 10 min. For dehydration, an ethanol series comprising 30%, 50%, 60%, 80%, 90%, 96%, and pure ethanol was applied. Samples were washed twice with the corresponding ethanol concentration and finally remained for 10 min in each solution. After this procedure, a 50:50 hexamethyldisilazane (HMDS)–ethanol solution was applied for 30 min followed by 100% HMDS for 30 min as a substitute for critical point drying. Afterwards, the samples were allowed to dry. SEM samples were sputter-coated with a 120°A layer of gold before examination by SEM (FEI Quanta 250 FEG, FEI Deutschland GmbH, Frankfurt, Germany).

### 2.12. Transmission Electron Microscopy (TEM)

Cells were grown on small discs of aclar foil (Science Services, Munich, Germany, #E50425-10) and fixed for 1 h in 2% Glutaraldehyde (Sigma-Aldrich, Taufkirchen, Germany, # G5882-100ML) with 2.5% Sucrose (Carl Roth, Karlsruhe, Germany, # 4621.1) and 3 mM CaCL2 (Sigma-Aldrich, Taufkirchen, Germany, # C7902-500G) in 0.1M HEPES buffer (Sigma-Aldrich, Taufkirchen, Germany # C7902-500G), pH 7,4. Samples were washed three times with 0.1M HEPES buffer and incubated with 1% Osmiumtetroxid (Science Services, Munich, Germany, # E19190) and 1% Potassium hexacyanoferrat (Sigma-Aldrich, Taufkirchen, Germany, # P8131) for 1 h at 4 °C. After 3 × 5 min wash with 0.1 M Cacodylate buffer (Applichem, Darmstadt, Germany, # A2140,0100), samples were dehydrated at 4 °C using ascending ethanol series (50%, 70%, 90%, 3 × 100%) for 7 min each. Infiltration was performed with a mixture of 50% Epon/ethanol for 1 h, 70% Epon/ethanol for 2 h, and with pure Epon (Science Services, Munich, Germany, # E14120) overnight at 4 °C. Samples were embedded into TAAB capsules (Agar Scientific, Rotherham, UK #G3744) and cured for 48 h at 60 °C.

Ultrathin sections of 70 nm were cut using an ultramicrotome UC6 (Leica Microsystems, Wetzlar, Germany) and a diamond knife (Diatome, Biel, Switzerland). Sections were stained with 1.5% uranyl acetate (Agar Scientific, # R1260A) for 15 min at 37 °C and with 3% Reynolds lead citrate solution made from lead (II) nitrate (Carl Roth, # HN32.1) and tri-sodium citrate dehydrate (Carl Roth, Karlsruhe, Germany, #4088.3) for 4 min. Images were acquired using a JEM-2100 Plus Transmission Electron Microscope (JEOL, Akishima, Japan) operating at 80 kV, equipped with a OneView 4K camera (Gatan, Pleasanton, CA, USA).

## 3. Results

**Figure 1:**
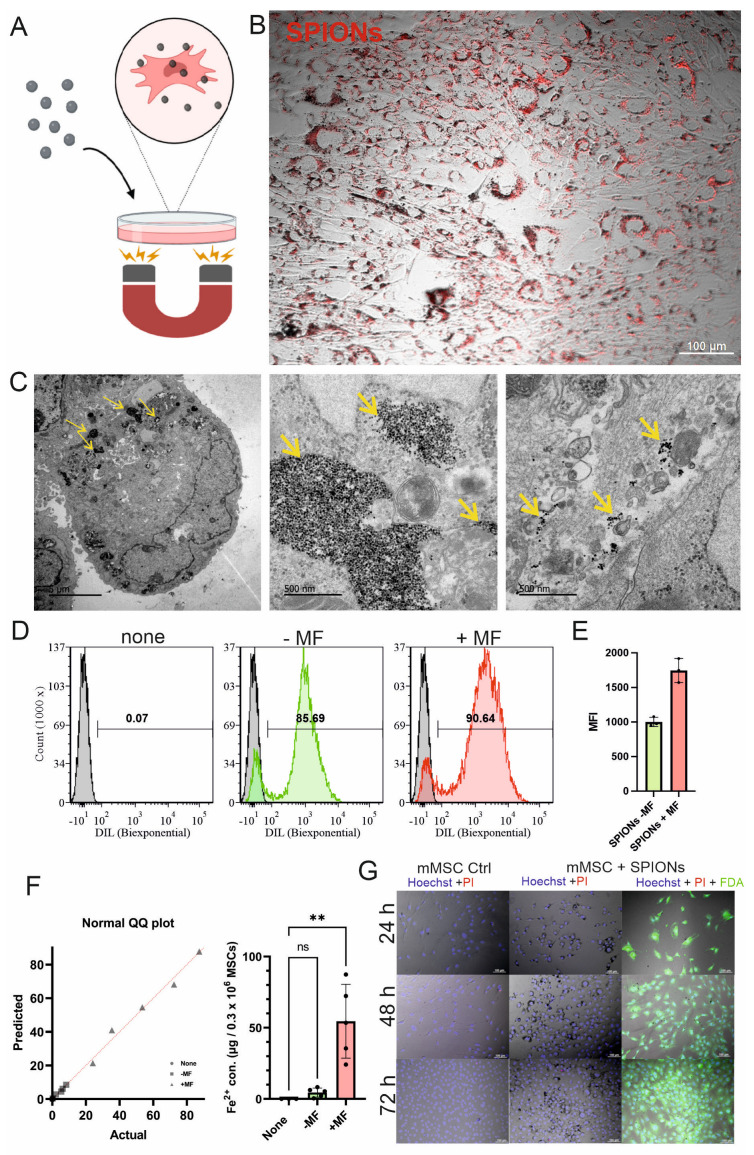
Concept of intracellular SPIONs loading. (**A**) The dish with plated mMSCs is placed on top of a magnet overnight. Medium is supplemented with 60 µg/mL SPIONs. (**B**) Fluorescence microscopic image of mMSCs loaded with Dil-labelled SPIONs; 10×. (**C**) TEM images of SPIONs-loaded mMSCs at 2000× and 25,000× magnification. SPIONs (arrows) were observed in cells in vesicles or inside the cytoplasm. (**D**) Flow cytometry of Dil-labelled SPIONs loaded intracellularly into mMSCs with (+MF) and without (−MF) magnet. (**E**) Mean fluorescence intensity (MFI) of SPIONs-loaded cells with (+MF) and without (−MF) a magnetic field. (**F**) Iron content of loaded mMSCs. Medium was supplemented with 120 µg SPIONs. Cells were loaded with SPIONs either without plate magnet (−MF) or with plate magnet (+MF) underneath the cell culture dish. Iron concentration was quantified through ferrozine assay and absorbance was measured at 562 nm. ** *p* < 0.05. (**G**) Cytotoxicity of SPIONs loaded intracellularly into mMSCs. Unloaded mMSC control in comparison with mMSCs loaded with SPIONs. Cell morphology was observed for 3 days and stained with Hoechst33342 and PI. SPIONs-loaded mMSCs were also stained with FDA. Scale bar: 100 µm.

**Figure 2:**
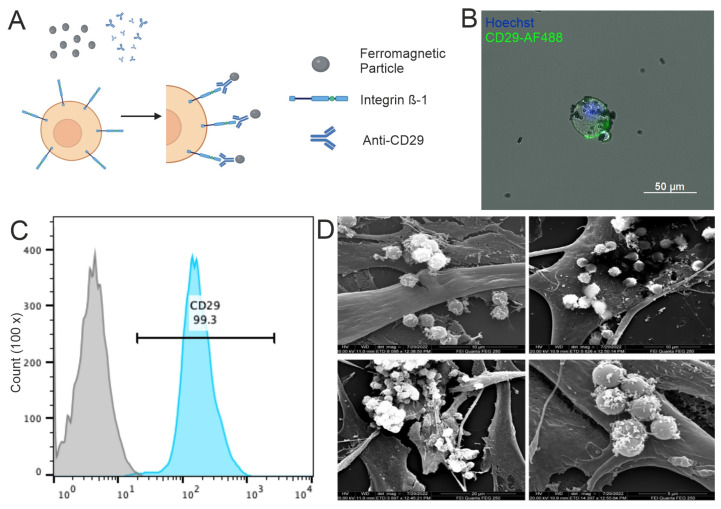
Ferromagnetic loading of mesenchymal stromal cells. (**A**) Concept of bio-linking ferromagnetic particles to the surface through anti-CD29 antibody. (**B**) Fluorescence microscopy of mMSC bio-linked to ferromagnetic particles. Cell nucleus is stained with Hoechst33342, Integrin ß-1 is stained with anti-CD29–Biotin–Streptavidin–Alexa Fluor 488; 20X. (**C**) Flow cytometry of unstained mMSCs control (grey) and mMSC stained with anti-CD29–Biotin–Streptavidin–Alexa Fluor 488. (**D**) SEM images of mMSCs bio-linked with ferromagnetic particles.

The feasibility of two methods for magnetically targeting MSCs is shown in Figure 1 and Figure 2. Firstly, the magnetic-force-mediated endocytosis of SPIONs into mMSCs was tested (Figure 1A) and confirmed with fluorescence microscopy (Figure 1B) and TEM, showing the intracellular locations of SPIONs inside the vesicles and solitarily in the cytoplasm (Figure 1C). Flow cytometry analysis revealed that, after 24 h of loading with a magnetic field (+MF) underneath, 90.64% of mMSCs had Dil-labelled SPIONs intracellularly. In contrast, after 24 h of loading without a magnetic field (−MF), 85.69% of mMSCs had Dil-labeled SPIONs inside the cells. Dil-labelled SPIONs were detected by fluorescence (Figure 1D). In flow cytometric measurements, the mean fluorescence intensity (MFI) of SPIONs-loaded cells shows that loading with a magnetic field (+MF) leads to double fluorescence intensity compared to cells loaded without a magnetic field (−MF) (n = 3), indicating a higher uptake of SPIONs per cell (Figure 1E).

To confirm this, the iron content of loaded mMSCs was measured using a ferrozine assay due to the availability of Fe^+2^ in the composition of the SPIONs. The measured iron concentration was normalized against an unloaded control group (none, only MSCs) (n = 3) and the iron content in µg/0.3 × 10^6^ cells was calculated. SPION-loaded mMSCs using magnetic-force-mediated endocytosis (+MF) contained 54.55 ± 25.90 µg iron oxide per 0.3 × 10^6^ cells (n = 5), compared to SPIONs-loaded mMSCs without using magnetic force (−MF) (n = 5), where the iron content was 4.46 ± 3.18 µg (Figure 1F). The iron concentrations of the unloaded MSC group and the loaded +MF group were significantly (*p* < 0.05) different.

The cytotoxic effect of SPIONs on mMSCs was tested for three days after the intracellular loading process (Figure 1G). To determine the live–dead ratio, mMSCs were stained with PI and FDA and the mortality of SPION-loaded MSCs was compared with the mortality of a control group; no difference could be observed.

Secondly, bio-linking ferromagnetic particles to the surface receptors of mMSCs using antibodies for magnetic targeting was shown (Figure 2A). Fluorescence microscopy (Figure 2B) demonstrated the successful bio-linking of ferromagnetic particles with antibodies. Flow cytometry with antibodies against CD29 verified the existence of an integrin- ß1 surface receptor on the cell membrane of the mMSC (Figure 2C). Scanning electron microscopy confirmed the location of ferromagnetic particles on the cell membrane (Figure 2C).

**Figure 3:**
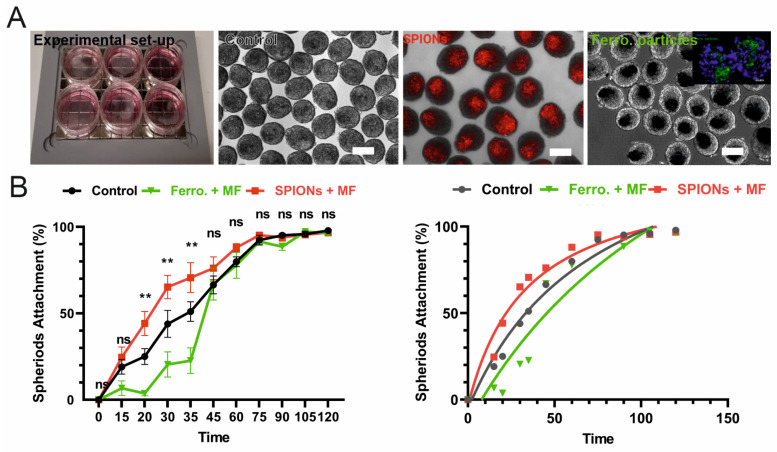
Adhesion experiment of magnetically targeted cellular spheroids on collagen-coated plates. (**A**) Experimental setups and morphologies of different spheroids. SPIONs (Dil-labelled, red colour)-loaded and ferromagnetic particle (Anti-CD29-AlexaFlour 488; green) co-incubated spheroids were visualised by fluorescence microscopy. Scale bars: 100 µm. (**B**) Attachment ratio was quantified at different time points. Left panel: experimental data. Right panel: mathematical curve fitting. Data are represented as mean ± SD. ** *p* < 0.05.

To compare the adhesion speed of mMSC spheroids on a collagen-I-coated plastic dish, mMSCs were loaded with SPIONs. Alternatively, ferromagnetic particles were bio-linked to CD29 cell surface receptors. Cell spheroids were generated by the hanging drop method. Spheroids were examined by transmission light and fluorescence microscopy showing control (no particles; colourless), SPION-labelled (Dil-labelled; red), and ferromagnetic-particle-labelled (CD29-linked Ferro; green) mMSC spheroids (Figure 3A). Next, the attachment of the mMSC spheroids was quantified at eleven time points during a total observation time of 120 min (n = 8). Statistical analysis showed that the first 45 min is the critical time window for mMSC spheroids adhesion. SPIONs significantly (*p* < 0.05) reduced the mMSC spheroids’ adhesion time in comparison with the control and Ferro (Figure 3B).

**Figure 4:**
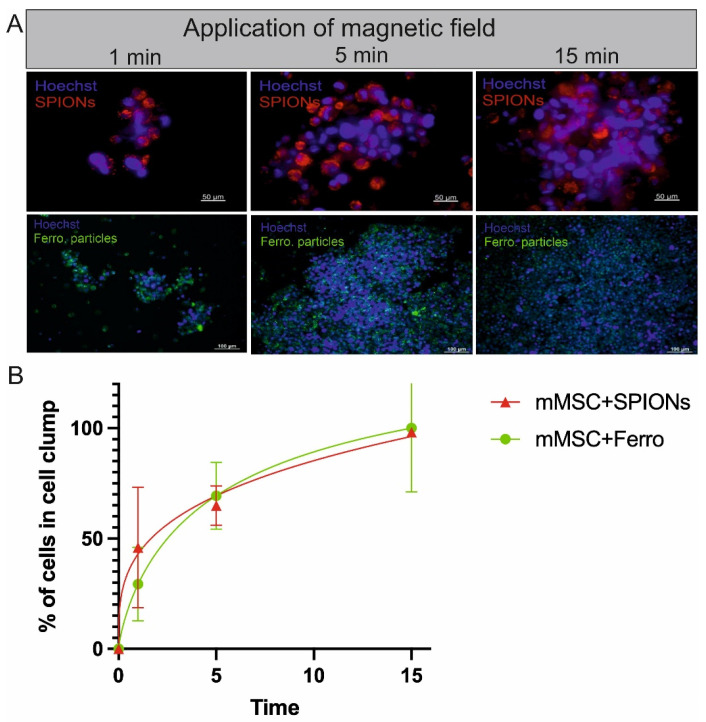
Magnetically induced cell clumping. (**A**) A magnet with a conic iron tip is placed under the dish harbouring the magnetically labelled cells, either labelled with SPIONs or with ferromagnetic particles, for 1, 5, and 15 min, and transferred to a slide for microscopy. Cells were stained with Hoechst33342. Cell clumps were imaged by fluorescence microscopy. (**B**) Quantification of cell count in magnetically induced clumps based on DNA content.

Next, we investigated the cell clustering and/or cell aggregation through magnetic force. SPION-labelled mMSCs (group 1) or ferromagnetic-particle-labelled mMSCs (group 2) were placed close to a magnet with a conic iron tip. The formation of cell clusters was observed and the cell number per cluster was determined through DNA quantification. The application of magnetic force was performed for 1, 5, and 15 min and the effect of cell clumping was observed by fluorescence microscopy (Figure 4A). DNA was isolated from the induced cell clumps for indirect cell quantification (Figure 4B). The percentage of cells within the cell clumps was calculated by dividing the calculated cell number after DNA OD measurement by the total cell number of the experiment. After 15 min, 100.00 ± 28.84% of mMSCs were found in the cell aggregate for ferromagnetic-particle-labelled cells and 98.12 ± 0.38% for SPIONs-loaded cells. Cell aggregates of SPION-labelled mMSCs macroscopically followed a permanent magnet (Video S1). When placed in the magnetic field of the permanent magnet (1 h), the aggregates formed a stable agglomerate (Video S2).

## 4. Discussion

Magnetic targeting has the potential to increase cell retention in different settings of cell replacement therapy. This is especially useful in applications where cell engraftment is critical, for example, in cardiac cell replacement therapy. In a previous study, Ottersbach and colleagues demonstrated the potential of magnetically targeting murine pluripotent stem-cell-derived cardiomyocytes (PSC-CMs) and murine embryonic cardiomyocytes (eCMs) to the myocardium [18]. Placing a magnet close to the injection site for 10 min was found to be sufficient for increasing cell retention by about 7-fold in the two weeks following the injection. Longer exposure to the magnet did not result in further improvement.

These data demonstrate the potential of magnetic targeting to increase cell engraftment in the heart. Although promising, the absolute cell retention remained low: when 200,000 PSC-CMs were transplanted, magnetic targeting increased the absolute number of engrafted CMs from 653 ± 34 to 4453 ± 724 cells 2 weeks after transplantation.

In a previous study, we showed that monocultures of murine PSC-CMs hardly engraft when transplanted into an acutely injured murine myocardium [11]. Real-time PCR-based quantification of engrafted PSC-CMs revealed a cell persistence of only 0.8% one day after transplantation. Interestingly, the engraftment rate remained the same when clumps of PSC-CMs were transplanted instead of single cell suspensions. In contrast, the generation of clumps from mouse bone marrow stromal cells and PSC-CMs resulted in a substantial increase in cell retention that outcompetes the previously discussed effect of magnetic targeting. It can be speculated that both methods, i.e., the formation of clusters of PSC-CMs with mMSC and the application of magnetic cell targeting, may act synergistically to enhance cell engraftment. Therefore, we use spherical clusters of mMSCs in this study to analyse the effect of magnetic forces on mMSC adhesion in vitro, and to pave the way for improved experimental designs for future pre-clinical experiments in vivo.

SPIONs were used to magnetically label mMSCs. For the adhesion experiments, clusters of mMSCs were prepared. The results show significantly faster cell adhesion when clusters are exposed to a magnetic field. More than 50% of the cell spheres attached within 30 min. We also employed a novel technology and labelled mMSCs with ferromagnetic particles that were hooked up to the cell membrane by antibodies against CD29 but could not demonstrate faster adhesion of the loaded mMSC clusters when exposed to a magnetic field.

These data suggest that faster attachment of cells to the extracellular matrix contributes to the enhancement effects of magnetic targeting when SPIONs are employed to load cells. On the other hand, it is possible that the effect is also due to forced cell aggregation. By using cell-surface-bound ferromagnetic particles, we aimed to generate a system were the short application of a strong magnetic field results in the permanent magnetisation of the particles resulting in the formation of forces between the labelled cells, even in the absence of the external magnet. The basic idea of this approach is to generate ferromagnetically labelled cells that can be rapidly magnetised by an external field and can result in the mechanically stable formation of aggregates.

We could show that there is a trend towards the faster attachment of SPION-loaded MSC spheroids. MSC clusters loaded on the surface with ferromagnetic particles attach more slowly; the presence of ferromagnetic cells on the cell membrane could be a possible explanation for this. To assess the aggregation of cells, SPION-labelled and ferromagnetic-particle-labelled cells were used, and cell aggregation was measured. In both experiments, aggregation was efficient and cell numbers in aggregates did not point to a more pronounced effect when ferromagnetic particles were used. Taking into account the fact that SPIONs from ferric oxide did not show toxic effects on the cells, loading with SPIONs seems favourable as compared to loading with ferromagnetic particles; this is because the ferromagnetic particles in this study contained chromium dioxide, and large extracellular particles may cause unforeseen effects in the myocardium. Moreover, uses of iron-oxide-based SPIONs have already advanced to clinical uses, showing minimal toxicity (for a review, see [21]).

With respect to the potential mechanism of magnetic targeting, we found that the adhesion of mMSCs to the matrix takes places in less than an hour, with more than 50% of the cells being attached after 30 min in the presence of a magnetic force. In contrast, the aggregation of cells in suspension appears faster, reaching 80% saturation within only 10 min. This finding points to the conclusion that the effects of increased cardiomyocyte engraftment observed by Ottersbach and colleagues is due to forced cell aggregation, because the application of the magnetic field for 10 min was sufficient for reaching the maximal effect.

In conclusion, magnetic targeting is a potential method for ensuring controlled cell aggregation, thus reducing the washout of transplanted cells. This has significant implications for more effective stem cell and cell replacement therapies. In future in vivo studies, we will investigate whether magnetic targeting acts synergistically with microtissue transplantation and improves cell retention after intramyocardial cell transplantation.

## Data Availability

Data is available on request.

## Supplementary Materials

The following supporting information can be downloaded at: https://www.mdpi.com/article/10.3390/bioengineering12060657/s1, Video S1: Movement of SPION labelled MSC spheroids along magnetic field gradients. Video S2: Formation of macroscopic assemblies of MSC spheroids following 1 h incubation in the magnetic field.
